# Constitutively Active Stat5b Expression in Dendritic Cells Enhances Treg-Mediated Elimination of Autoreactive CD8^+^ T Cells in Autoimmune Diabetes

**DOI:** 10.3390/ijms27020794

**Published:** 2026-01-13

**Authors:** Puregmaa Khongorzul, Farhan Ullah Khan, Daphnée Levasseur, Denis Gris, Abdelaziz Amrani

**Affiliations:** 1 Department of Pediatrics, Immunology Division, Faculty of Medicine and Health Sciences, Centre de Recherche du CHUS, Université de Sherbrooke, 3001, 12th Avenue North, Sherbrooke, QC J1H 5N4, Canada; puregmaa.khongorzul@usherbrooke.ca (P.K.); farhan.ullah.khan@usherbrooke.ca (F.U.K.); daphnee.levasseur@usherbrooke.ca (D.L.); 2 Department of Pharmacology-Physiology, Faculty of Medicine and Health Sciences, Centre de Recherche du CHUS, Université de Sherbrooke, 3001, 12th Avenue North, Sherbrooke, QC J1H 5N4, Canada

**Keywords:** type 1 diabetes, regulatory T cells, immunity, tolerance, Stat5

## Abstract

In type 1 diabetes (T1D) in non-obese diabetic (NOD) mice, dendritic cells (DCs) exhibit a *Stat5b* mutation that impairs regulatory T cell (Tregs) numbers and suppressive function. To correct this defect, we generated transgenic NOD mice expressing constitutively active Stat5b (NOD.Stat5b-CA) in DCs, which conferred protection from diabetes that was associated with an expanded Treg population and a marked reduction in CD8^+^ T cell frequencies in secondary lymphoid organs. However, the phenotypic characteristics and underlying mechanisms to eliminate CD8^+^ T cells in NOD.Stat5b-CA mice are unknown. In this study, we found that the frequency of Tregs was significantly higher in the thymus and peripheral lymphoid organs of NOD.Stat5b-CA mice compared with NOD mice. Tregs in the peripheral lymphoid organs exhibited increased expression of activation markers CD69 and OX40, alongside reduced CD62L. We also found that CD8^+^ T cell frequencies were reduced in the peripheral organs but not in the thymus of NOD.Stat5b-CA mice, while CD4^+^ T cell frequencies remained unchanged across all organs. Furthermore, NOD.Stat5b-CA mice exhibited a reduced frequency of central Tregs (CD62L^high^ CD44^low^) and increased frequency of effector Tregs (CD62L^low^ CD44^high^) under steady-state conditions compared to NOD mice. Notably, Tregs from NOD.Stat5b-CA mice displayed enhanced cytotoxic activity, evidenced by increased expression of perforin, granzyme B, and Fas ligand, potentially mediating CD8^+^ T cell frequency reduction. Collectively, these findings highlight a novel role for Stat5b-CA.DC-educated Tregs in modulating immune responses by eliminating peripheral pathogenic CD8^+^ T cells via cytotoxic pathways, thereby contributing to immune regulation in NOD.Stat5b-CA mice.

## 1. Introduction

Regulatory T cells (Tregs) are a specialized subset of CD4^+^ T cells essential for maintaining immune tolerance and preventing excessive immune responses. They are characterized by the expression of the IL-2 receptor α-chain (CD25) and the master transcription factor forkhead box protein P3 (Foxp3), which governs their immunosuppressive function [1,2]. Tregs play a central role in maintaining immune tolerance and preventing autoimmunity by suppressing autoreactive effector T-cell activities [3]. Multiple studies have shown that defects in either the number or the function of Tregs are implicated in the breakdown of self-tolerance, leading to autoimmune diseases, including type 1 diabetes (T1D) [4,5,6].

T1D is an autoimmune disorder wherein pancreatic β cells are targeted and destroyed by autoreactive CD4^+^ and CD8^+^ T cells [7]. CD4^+^ T cells are required for diabetes development [8]. Indeed, Th1 cells can transfer diabetes to young NOD mice [9]. Similarly, CD4^+^ T cell clones specific for InsB9-23 can cause diabetes in adoptive transfer models in lymphopenic mice [10,11,12]. Recent work has shown that islet-antigen-specific CD4^+^ T cells that recognize Hybrid-insulin C-chromogranin A (InsC-ChgA) peptide skewed toward a distinct T helper type 1 (Th1) effector phenotype are pathogenic upon transfer into NOD.RAG1^−/−^ mice [13]. The same work also showed that transfer of 8.3-CD8^+^ T cells to NOD.RAG1^−/−^ mice did not transfer diabetes on their own. These studies suggest that pathogenic CD8^+^ T cells require CD4^+^ T cell help to cause diabetes, whereas CD4^+^ Th1 cells that recognize hybrid insulin peptide are highly pathogenic and can independently cause diabetes. These data are in agreement with a previous work showing that highly diabetogenic CD4^+^ T cells from TCRαβ transgenic 4.1-NOD mice can cause diabetes in the absence of CD8^+^ T cells, as demonstrated by full diabetes onset in 4.1-NOD.RAG2^−/−^ mice and full development of diabetes in TCRαβ transgenic 8.3-NOD mice and reduction in diabetes by 50% in 8.3-NOD RAG2^−/−^ [14,15]. Therefore, the assistance of CD4^+^ T cells is necessary for diabetogenic CD8^+^ T cells (like 8.3-CD8^+^ T cells) to acquire optimal effector function. In the context of T1D, the non-obese diabetic (NOD) mouse model has been extensively used to study the pathogenesis and potential interventions for the disease [16,17,18]. Several studies have reported that NOD mice exhibit significant defects in Treg frequency, function, and stability, which are primarily attributed to impaired dendritic cells (DCs) function [19,20]. DCs play a pivotal role in antigen presentation and the induction of Tregs [21]. Emerging evidence highlights that DCs in NOD mice have a *Stat5b* mutation affecting the DNA-binding domain, leading to impaired Stat5b DNA binding and reduced expression of downstream gene expression involved in immunoregulation [22]. The *Stat5b* mutation in NOD mice compromises the tolerogenic function of DCs, impairing their ability to generate effective Tregs. To tackle this issue, we generated a transgenic NOD mouse model in which DCs express a constitutively active form of the *Stat5b* gene (NOD.Stat5b-CA mice). Our preliminary findings indicated that active Stat5b expression in DCs re-established their tolerogenic properties [23,24] and enabled DCs to re-educate Tregs and sustain long-term immune tolerance, thereby protecting against T1D [25]. Notably, a significant reduction in the proportion of CD8^+^ T cells, approximately 50%, was observed in the peripheral lymphoid organs of NOD.Stat5b-CA mice compared to NOD mice, while the proportion of CD4^+^ T cells remained unaltered and was skewed toward the Th2 phenotype [25]. Despite these promising findings, the underlying mechanisms responsible for the reduction in CD8^+^ T cell frequency in NOD.Stat5b-CA mice remained unexplored.

Tregs modulate immune responses by suppressing the activation and proliferation of effector immune cells, including CD4^+^ and CD8^+^ T cells, through multiple mechanisms [26]. These include the production of immunomodulatory cytokines like interleukin-10 (IL-10), IL-35, and transforming growth factor-beta (TGF-β), which suppress immune cell activity [26]. Tregs can also induce apoptosis in effector T cells through IL-2 deprivation, leading to Bim-mediated apoptosis [27], and express CD39 and CD73, which convert extracellular adenosine triphosphate (ATP) to adenosine, further inhibiting effector T cell function [28,29]. Additionally, Tregs can directly inhibit DC function through CTLA-4 and LAG-3, reducing the expression of co-stimulatory molecules and inducing the production of immunosuppressive metabolites, such as indoleamine 2,3-dioxygenase (IDO), which leads to T cell anergy and apoptosis [30]. Moreover, another less-studied mechanism utilized by Tregs is their cytolytic activity, which includes the granzyme/perforin and Fas/FasL pathways, both of which induce apoptosis in target cells. In 2004, Grossman et al. first reported that human CD4^+^ thymic Tregs (tTregs) express granzyme A, while peripheral CD4^+^ Tregs (pTregs) express granzyme B [31]. These granzyme-expressing CD4^+^ Treg subtypes were found capable of eliminating autologous target cells in a perforin-dependent but TCR-independent manner [32]. The same study, also revealed that activated T cells and immature DCs were more susceptible to preferential elimination compared to resting T cells and mature DCs, indicating that not all target cells exhibit equal susceptibility to CD4^+^ Treg-mediated killing [32]. In addition, Tregs express Fas ligand (FasL) and can induce apoptosis in target cells via Fas/FasL interactions. Particularly, Tregs expressing Fas ligand (FasL) has been shown to selectively induce apoptosis in CD8^+^ T cells within tumor microenvironments, including head and neck squamous cell carcinoma (HNSCC) and hepatocellular carcinoma (HCC) [24,25]. These findings suggest that Treg-mediated cytotoxicity may serve as a potent mechanism for regulating CD8^+^ T cell homeostasis.

Given these established cytolytic functions of Tregs, we hypothesized that the Stat5b-CA.DC educated Tregs in NOD.Stat5b-CA mice might contribute to the reduction in CD8^+^ T cells by eliminating them through granzyme/perforin and Fas/FasL-mediated pathways. Our study provides compelling evidence supporting that Tregs from NOD.Stat5b-CA mice produce significantly higher levels of perforin and granzyme and exhibit elevated expression of FasL compared to Tregs from NOD mice. Furthermore, these Tregs displayed enhanced suppressive capacity of CD8^+^ T cell proliferation by inducing a high rate of CD8^+^ T cell apoptosis.

## 2. Results

### 2.1. CD8^+^ T Cell Frequency Is Reduced in Peripheral Lymphoid Organs but Not in the Thymus of NOD.Stat5b-CA Mice

**Figure 1:**
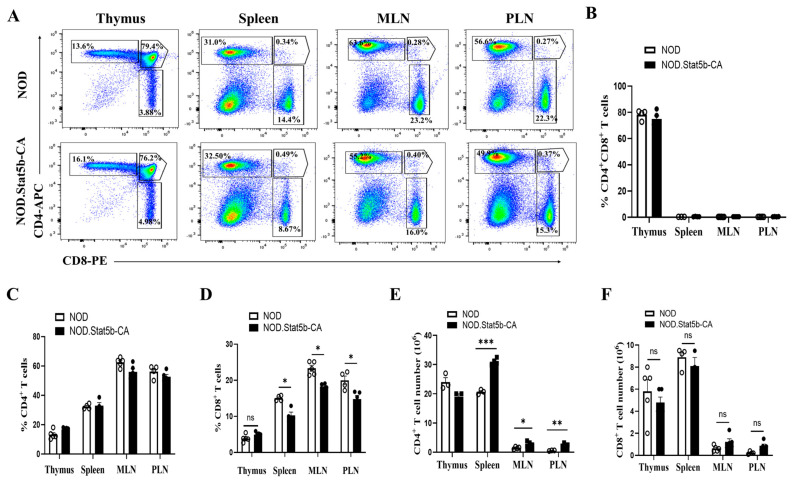
The frequency of CD8^+^ but not CD4^+^ T cells is reduced in peripheral lymphoid organs of NOD.Stat5b-CA mice. (**A**) Representative FACS profile of double-positive (DP; CD4^+^CD8^+^), CD4^+^ and CD8^+^ T cell frequencies in the thymus, spleen, mesenteric lymph nodes (MLN), and pancreatic lymph nodes (PLN) from NOD and NOD.Stat5b-CA mice. (**B**–**D**) Bar graphics showing the average frequencies of DP, CD4^+,^ and CD8^+^ T cells in the thymus and spleen, MLN, and PLN of NOD and NOD.Stat5b-CA mice. (**E**,**F**) Bar graphics showing the absolute number of CD4^+^ and CD8^+^ T cells in the thymus and spleen, MLN, and PLN of NOD and NOD.Stat5b-CA mice. Error bars indicate the mean ± SEM of four-five independent experiments (*n* = 4) with 5 mice per group. *p* values were calculated using Student’s *t*-test. ns, not significant; * *p* ˂ 0.05; ** *p* ˂ 0.01; *** *p* ˂ 0.001.

Previously, we examined the impact of Stat5b-CA.DCs on CD4^+^ and CD8^+^ T cells homeostasis and immune response. Our findings revealed that the frequency of CD4^+^ T cells remained unaltered, while the frequency of CD8^+^ T cells was significantly reduced (approximately 50%) in the peripheral lymphoid organs of NOD.Stat5b-CA mice compared to NOD mice [25]. However, the underlying mechanism responsible for this reduction in CD8^+^ T cell frequency in NOD.Stat5b-CA mice remain unclear. The decrease in CD8^+^ T cell frequency could result from negative selection in the thymus or in the periphery through several mechanisms, such as anergy, deletion via apoptosis, and/or immune regulation. Therefore, we investigated the frequencies of double-positive (DP; CD4^+^CD8^+^), CD4^+,^ and CD8^+^ T cells in the thymus and various peripheral lymphoid organs of NOD and NOD.Stat5b-CA mice. We found that the frequencies of DP and CD4^+^ T cells were unchanged in both the thymus and peripheral lymphoid organs (spleen, MLN, and PLN) of NOD and NOD.Stat5b-CA mice (Figure 1A–C). In contrast, the frequency of CD8^+^ T cells was significantly reduced in the spleen, PLN, and MLN of NOD.Stat5b-CA mice compared to NOD mice, while no difference was found in the thymus of both strains of mice (Figure 1A,D). Furthermore, we also evaluated the absolute number of CD4^+^ and CD8^+^ T cells in all four organs. Data showed that there was no prominent difference in the total number of CD4^+^ T cells in the thymus of NOD and NOD.Stat5b-CA mice, while a significant difference was noticed in the peripheral lymphoid organs (Figure 1E). Notably, we did not observe any obvious difference in the absolute number of CD8^+^ T cells in all four organs among these two strains of mice (Figure 1F). These findings suggest a reduction in CD8^+^ T cell frequency in NOD.Stat5b-CA mice that are attributable to the mechanisms of immune regulation in the periphery rather than in the thymus.

### 2.2. NOD.Stat5b-CA Mice Exhibit Increased Treg Frequency and Elevated Foxp3 Expression in the Thymus and Lymphoid Organs

**Figure 2:**
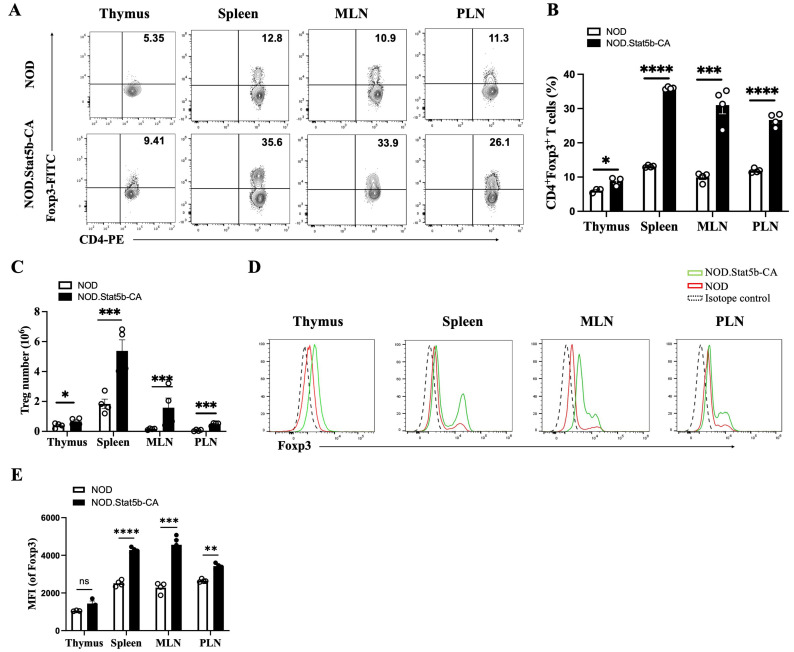
Increased frequency and Foxp3 expression in Tregs from NOD.Stat5b-CA mice. (**A**) Representative FACS profile of CD4^+^Foxp3^+^ T cells in the thymus, spleen, MLN, and PLN from NOD and NOD.Stat5b-CA mice. (**B**) Bar graphs showing CD4^+^Foxp3^+^ T cell frequencies in the CD4^+^ T cell population in the thymus, spleen, MLN, and PLN of 4 to 6-week-old NOD and NOD.Stat5b-CA mice. (**C**) Absolute number of Treg cells in the thymus, spleen, MLN, and PLN of NOD and NOD.Stat5b-CA mice. (**D**) Representative histograms of Foxp3 expression in Tregs in the thymus, spleen, MLN, and PLN from NOD and NOD.Stat5b-CA mice. (**E**) Bar graph showing the quantification of Mean Fluorescence Intensity (MFI) values for Foxp3 expression in Tregs in NOD and NOD.Stat5b-CA mice. Error bars indicate the mean ± SEM of three-four independent experiments (*n* = 4). *p*-values were calculated using Student’s *t*-test. ** *p* ˂ 0.01; *** *p* ˂ 0.001; **** *p* ˂ 0.0001.

Previous studies have shown that Tregs from HNSCC patients selectively induce the apoptosis of autologous CD8^+^ T cells but not CD4^+^ T cells [33]. This observation led us to explore whether the reduction in CD8^+^ T cells is associated with Treg expansion in NOD.Stat5b-CA mice. Therefore, we analyzed the frequency and the expression of Foxp3 in Tregs in the thymus and peripheral lymphoid organs of NOD and NOD.Stat5b-CA mice. FACS analysis data showed that NOD.Stat5b-CA mice had a significantly higher frequency of CD4^+^Foxp3^+^ T cells in the thymus, spleen, MLN, and PLN than in NOD mice (Figure 2A,B). Additionally, there was a significant increase in the total cell number of CD4^+^Foxp3^+^ T cells in all lymphoid organs of NOD.Stat5b-CA compared to NOD mice (Figure 2C). Moreover, Tregs from NOD.Stat5b-CA exhibited higher levels of Foxp3 expression than those from NOD mice across all four organs (Figure 2D,E). These results collectively indicate that NOD.Stat5b-CA mice harbor a greater number of Tregs with heightened Foxp3 expression levels in the thymus and peripheral lymphoid organs than NOD mice. Furthermore, these data suggest that the rise in high Foxp3-expressing Tregs observed in NOD.Stat5b-CA mice are associated with a decrease in CD8^+^ T cells in peripheral tissues but not in the thymus.

### 2.3. Tregs from NOD.Stat5b-CA Mice Display an Activated Phenotype

**Figure 3:**
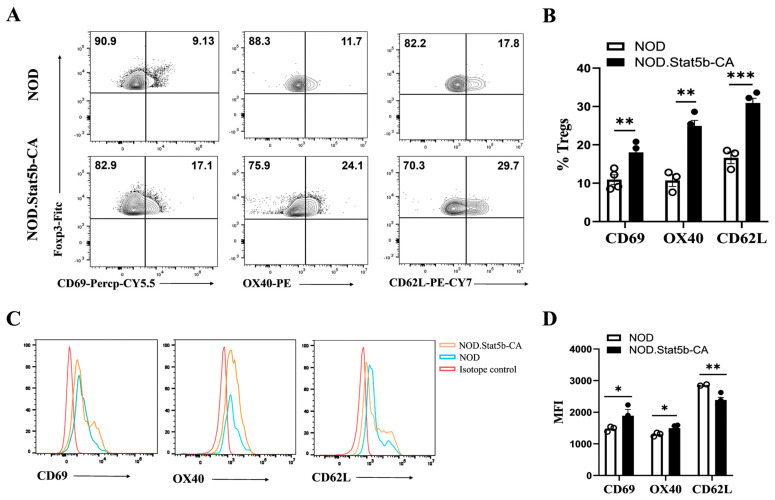
Tregs of NOD.Stat5b-CA mice exhibit an activated phenotype. (**A**,**B**) Dot plots and bar graphs showing Foxp3^+^CD69^+^, Foxp3^+^OX40^+,^ and Foxp3^+^CD62L^+^ cell frequencies among the CD4^+^Foxp3^+^ T cell population in the spleen of NOD and NOD.Stat5b-CA mice. (**C**,**D**) Representative histograms (left side) and quantification of Mean Fluorescence Intensity (MFI) values (right side) showing CD69, OX40, and CD62L expression levels in CD4^+^Foxp3^+^ Tregs in NOD and NOD.Stat5b-CA mice. Error bars indicate the mean ± SEM of three-four independent experiments (n = 4). *p*-values were calculated using Student’s *t*-test. * *p* ˂ 0.05; ** *p* ˂ 0.01; *** *p* ˂ 0.001.

Treg are known to adopt a naïve phenotype and require either in vitro or in vivo activation to acquire their full potential suppressive function [34]. To investigate whether the differences in Foxp3 expression between Tregs of NOD and NOD.Stat5b-CA mice impact their activation status, we evaluated Treg-associated activation markers in the thymus and various lymphoid organs. Flow cytometry (FACS) analysis revealed that a significantly higher frequency of CD4^+^Foxp3^+^ Tregs expressing CD69, OX40, and CD62L were present in the thymus, spleen, MLN, and PLN of NOD.Stat5b-CA mice compared to NOD mice (Figure 3A,B and Supplementary Figure S1A–F). Moreover, CD4^+^Foxp3^+^ Tregs from NOD.Stat5b-CA mice exhibited elevated levels of CD69 and OX40 compared to CD4^+^Foxp3^+^ Tregs from NOD mice (Figure 3C,D and Supplementary Figure S1G–L). However, CD62L expression on CD4^+^Foxp3^+^ Tregs was similar in the thymus of both mouse strains (Supplementary Figure S1G,H). Interestingly, CD4^+^Foxp3^+^ Tregs from NOD.Stat5b-CA mice expressed lower levels of CD62L in the spleen, MLN, and PLN compared to NOD mice (Figure 3C,D and Supplementary Figure S1I–L). These results indicate that CD4^+^Foxp3^+^ Tregs from NOD.Stat5b-CA mice are more activated than CD4^+^Foxp3^+^ Tregs from NOD mice. However, no difference in CD62L expression in the thymus between the two strains suggests that the CD4^+^Foxp3^+^ Tregs of NOD.Stat5b did not acquire a full activation status in the thymus.

### 2.4. NOD.Stat5b-CA Mice Have a High Frequency of Effector Tregs and a Lower Frequency of Central Tregs Under Steady-State Conditions

**Figure 4:**
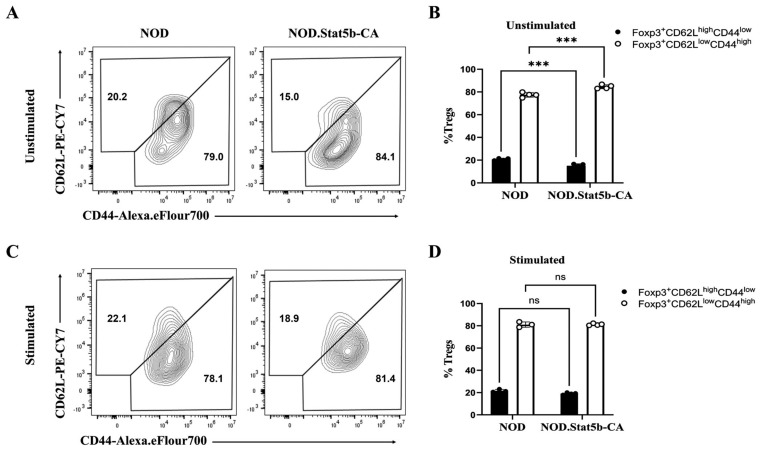
The proportion of CD62L^high^CD44^low^ and CD62L^low^CD44^high^ Tregs in NOD.Stat5b-CA and NOD mice. Splenic cells from NOD and NOD.Stat5b-CA mice were left unstimulated or stimulated with anti-CD3 (5 μg/mL), anti-CD28 (2 μg/mL), and IL-2 (20 U/mL) for 24 h. After that, cells were collected, washed, and stained with the corresponding appropriate fluorescent-conjugated antibodies. (**A**–**C**): Representative FACS data of CD62L^high^CD44^low^ and CD62L^low^CD44^high^ Tregs (gated on CD4^+^Foxp3^+^ cells; CD4-PE and Foxp3-Fitc) frequency in the spleens of NOD and NOD.Stat5b-CA mice. (**B**–**D**) Bar graphics showing the percentages of CD62L^high^CD44^low^ and CD62L^low^CD44^high^ Tregs in the spleens of NOD and NOD.Stat5b-CA mice. Error bars indicate the mean ± SEM of four independent experiments (n = 4). *p*-values were calculated by one-way ANOVA followed by Tukey’s post hoc test. ns, not significant; *** *p* ˂ 0.001.

Previous studies have shown that Tregs can be categorized into central Tregs (cTregs) and effector Tregs (eTregs) based on their activation status as determined by the expression of specific cell surface markers. In mice, cTregs are defined by a CD62L^high^ CD44^low^ phenotype, whereas eTregs exhibit a CD62L^low^ CD44^high^ phenotype [35]. Since peripheral Tregs of NOD.Stat5b-CA mice showed a highly activated status compared to peripheral Tregs of NOD mice, we sought to investigate eTreg and cTreg frequencies in the spleens of both strains of mice before and after anti-CD3/CD28 and IL-2 stimulation. FACS data revealed that unstimulated splenic cells of NOD.Stat5b-CA mice comprise a lower frequency of CD62L^high^CD44^low^ cTregs and a higher frequency of CD62L^low^CD44^high^ eTregs compared to the NOD mice (Figure 4A,B). However, under stimulation, no significant differences were observed in CD62L^high^ CD44^low^ cTregs or CD62L^low^ CD44^high^ eTregs frequencies between the two groups of mice (Figure 4C,D). These results indicate that NOD.Stat5b-CA mice have a reduced frequency of splenic cTregs and an increased frequency of splenic eTregs under steady-state conditions compared to NOD mice.

### 2.5. Tregs of NOD.Stat5b-CA Mice Express Higher Levels of Perforin and Granzyme B than Tregs of NOD Mice

**Figure 5:**
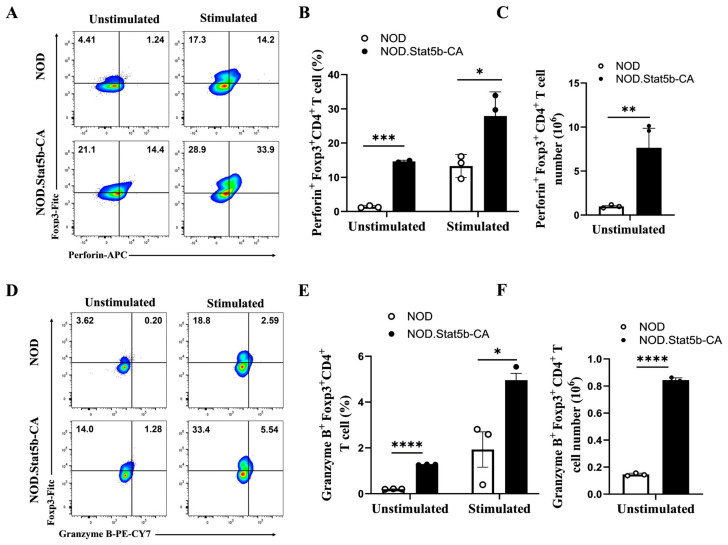
Tregs of NOD.Stat5b-CA mice express higher levels of perforin and granzyme B. Splenic cells from NOD and NOD.Stat5b-CA mice were left unstimulated or stimulated with anti-CD3 (5 μg/mL), anti-CD28 (2 μg/mL), and IL-2 (20 U/mL) for 24 h. Thereafter, cells were collected, washed, and stained with corresponding fluorescent-conjugated antibodies as described in material and methods. (**A**) Representative FACS data of perforin expression in Tregs (Foxp3^+^perforin^+^) among CD4^+^ T cells (CD4-PE conjugated) in the spleen of NOD and NOD.Stat5b-CA mice. (**B**,**C**) Percentage of perforin-expressing Tregs among CD4^+^ T cells and the absolute number of perforin^+^ CD4^+^Foxp3^+^ Treg cells in NOD and NOD.Stat5b-CA mice. (**D**) Representative FACS data of granzyme B expression in Tregs (Foxp3^+^ granzyme B^+^) among CD4^+^ T cells in the spleen of NOD and NOD.Stat5b-CA mice. (**E**,**F**) Percentage of granzyme B expressing Tregs among CD4^+^ T cells and the absolute number of granzyme B^+^ CD4^+^Foxp3^+^ T cells in NOD and NOD.Stat5b-CA mice. Error bars indicate the mean ± SEM of three independent experiments (n = 3). *p*-values were calculated using Student’s *t*-test. * *p* ˂ 0.05; ** *p* ˂ 0.01; *** *p* ˂ 0.001; **** *p* ˂ 0.0001.

It is known that Tregs suppress effector T cells through various mechanisms, including granzyme/perforin-dependent and Fas/FasL-dependent cytotoxicity [36]. Given the high frequency of activated eTregs in NOD.Stat5b-CA mice, we hypothesized that these eTregs might mediate the reduction of CD8^+^ T cells in a granzyme/perforin- and/or Fas/FasL-dependent manner. To this end, we evaluated perforin and granzyme B expression in splenic Tregs from both NOD.Stat5b-CA and NOD mice using flow cytometry. We found that Treg cells of NOD.Stat5b-CA mice expressed a significantly higher level of perforin compared to NOD mice under both unstimulated and stimulated conditions (Figure 5A,B). Additionally, there was a significant increase in the absolute number of perforin-expressing Treg cells in NOD.Stat5b-CA mice compared to NOD mice (Figure 5C). Similarly, Tregs of NOD.Stat5b-CA mice express higher levels of granzyme B compared to Tregs of NOD mice under both unstimulated and stimulated conditions (Figure 5D,E), along with a significant increase in the absolute number of granzyme B^+^ Treg cells (Figure 5F). Altogether, these data indicate that splenic cells of NOD.Stat5b-CA mice contain a higher frequency of Treg cells expressing elevated levels of granzyme B- and perforin compared to NOD mice.

### 2.6. Tregs from NOD.Stat5b-CA Mice Express Elevated Levels of FAS Ligand

**Figure 6:**
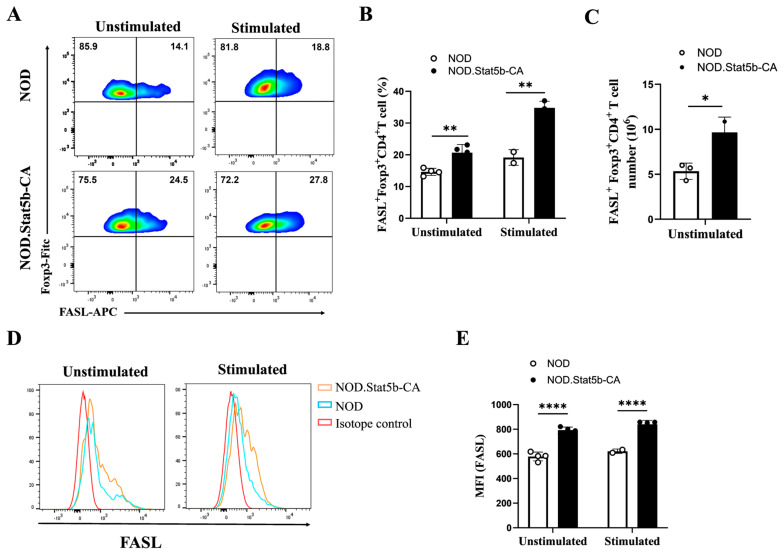
Tregs of NOD.Stat5b-CA expresses elevated levels of FASL. Splenic cells from NOD and NOD.Stat5b-CA mice were left unstimulated or stimulated with anti-CD3 (5 μg/mL), anti-CD28 (2 μg/mL), and IL-2 (20 U/mL) for 24 h. Thereafter, cells were collected, washed, and stained with corresponding fluorescent-conjugated antibodies as described in material and methods. (**A**) Representative FACS data of FASL^+^ CD4^+^Foxp3^+^ Treg cells in the spleen of NOD and NOD.Stat5b-CA mice. (**B**,**C**) Percentages and absolute numbers of FASL-expressing CD4^+^Foxp3^+^Treg cells in NOD and NOD.Stat5b-CA mice. (**D**) Representative histograms of FASL-expression in splenic CD4^+^Foxp3^+^ Tregs of NOD and NOD.Stat5b-CA mice. (**E**) Bar graph showing Mean Fluorescence Intensity (MFI) values for FASL expression levels in splenic CD4^+^Foxp3^+^ Tregs of NOD and NOD.Stat5b-CA mice. Error bars indicate the mean ± SEM of four independent experiments (n = 4). *p*-values were calculated using Student’s *t*-test. * *p* ˂ 0.05; ** *p* ˂ 0.01; **** *p* ˂ 0.0001.

Next, we examined the frequency and expression levels of FAS ligand (FASL) on Tregs of NOD.Stat5b-CA and NOD mice. FACS analysis data showed that under unstimulated conditions, spleen cells of NOD.Stat5b-CA mice exhibited a higher frequency of FASL^+^ CD4^+^Foxp3^+^ T cells compared to the spleens of NOD mice (Figure 6A,B). After stimulation, the frequencies of FASL^+^ CD4^+^Foxp3^+^ T cells were increased in both strains of mice but remained higher in the spleens of NOD.Stat5b-CA than in the spleens of NOD mice (Figure 6A,B). Additionally, there was a significant increase in the absolute number of FASL^+^ CD4^+^Foxp3^+^ T cells in NOD.Stat5b-CA mice compared to NOD mice (Figure 6C). Furthermore, the expression level of FASL in splenic Tregs of NOD.Stat5b-CA mice were significantly elevated compared to splenic Tregs of NOD mice before and after stimulation (Figure 6D,E). Collectively, these results indicate that Stat5b-CA expression in dendritic cells of NOD mice increased the frequency of high FASL-expressing Tregs.

### 2.7. Tregs of NOD.Stat5b-CA Mice Exhibit an Enhanced Capacity to Suppress Proliferation and Induce Apoptosis of Autoreactive CD8^+^ T Cells

**Figure 7:**
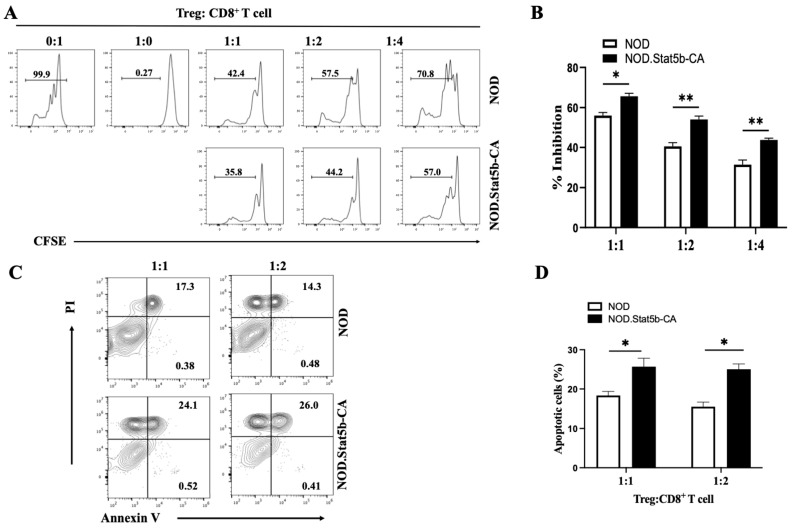
Tregs of NOD.Stat5b-CA mice exhibit a better ability to suppress CD8^+^ T cell proliferation and induce CD8^+^ T cell apoptosis compared to Tregs from NOD mice. (**A**) Tregs were purified from both NOD.Stat5b-CA and NOD mice and co-cultured with highly diabetogenic, CFSE-labeled 8.3-CD8^+^ T effector cells at ratios of 0:1, 1:0, 1:1, 1:2, and 1:4. These cells were then stimulated with anti-CD3 (5 μg/mL) and CD28 (2 μg/mL) antibodies. After 3 days of co-culture, CFSE dilution in 8.3-CD8^+^ T cells was assessed using flow cytometry. (**B**) The bar graph shows the percentage of proliferating 8.3-CD8^+^ T cells co-cultured with Tregs from NOD and NOD.Stat5b-CA mice at different ratios. (**C**) Flow cytometry analysis of PI and Annexin V staining was performed on 8.3-CD8^+^ T cells co-cultured with Tregs from both NOD and NOD.Stat5b-CA mice for 24 h at 8.3-CD8^+^ T cell: Treg ratios 1:1 and 1:2. (**D**) The bar graph illustrates the percentage of total apoptotic 8.3-CD8^+^ T cells co-cultured with Tregs from NOD and NOD.Stat5b-CA mice at different ratios. Apoptotic cells include both Annexin V^+^/PI^−^ and Annexin V^+^/PI^+^ cells. Error bars indicate the mean ± SEM of four independent experiments (n = 4). *p*-values were calculated using Student’s *t*-test. * *p* ˂ 0.05; ** *p* ˂ 0.01.

The above results showed that Tregs of NOD.Stat5b-CA were enriched with eTreg and, high expression of perforin/granzyme B and FasL, two mechanisms used to kill target cells by apoptosis. Therefore, we asked whether NOD.Sta5b-CA Tregs are also endowed with high suppressive function and apoptosis against effector CD8^+^ T cells, which we found reduced in NOD.Stat5b-CA mice. To this end, we used diabetogenic 8.3-CD8^+^ T effector cells expressing the TCRαβ receptor of islet-derived diabetogenic CD8^+^ T cell clones of NOD mice that contribute to beta cell destruction and diabetes development [37,38]. These 8.3-CD8^+^ T effector cells were consistently present in NOD islets, and with the help of CD4^+^ T cells, they transfer diabetes into NOD.Scid mice [13,39], and accelerate diabetes onset when highly expressed in NOD mice (8.3-NOD transgenic mice) [14]. Therefore, diabetogenic CFSE-labeled 8.3-CD8^+^ T cells were used as effector cells to assess Treg suppressive activities. For this purpose, purified Tregs from NOD.Stat5b-CA and NOD mice were co-cultured with anti-CD3 and anti-CD28-stimulated 8.3-CD8^+^ T cells. After 3 days of co-culture, inhibition of 8.3-CD8^+^ T cell proliferation was determined by flow cytometry. The results revealed that at a 1:1 Treg:CD8^+^ T cell ratio, Tregs from NOD.Stat5b-CA mice exhibited significantly greater suppressive capacity compared to Tregs from NOD mice (Figure 7A,B). Importantly, even increasing the number of 8.3-CD8^+^ T cells over Tregs (ratios 1:2 and 1:4), Tregs of NOD.Stat5b-CA mice continued to demonstrate a superior ability to suppress 8.3-CD8^+^ T cell proliferation compared to Tregs of NOD mice (Figure 7A,B). Next, we investigated whether Tregs from NOD and NOD.Stat5b-CA mice reduce CD8^+^ T cell proliferation through apoptosis. To this end, we co-cultured Tregs from NOD.Stat5b-CA and NOD mice with 8.3-CD8^+^ T effector cells for 8 h. Then cells were stained with anti-CD8 mAb, Annexin V, and PI and analyzed by flow cytometry. The results showed that Tregs from NOD.Stat5b-CA mice induced more 8.3-CD8^+^ T cell apoptosis compared to Tregs from NOD mice at all Treg/CD8^+^ T cell ratios (Figure 7C,D). Altogether, these results indicate that Tregs from NOD.Stat5b-CA mice exhibit significantly enhanced suppressive capacity against effector CD8^+^ T cells by both inhibiting their proliferation and promoting apoptosis, as compared to Tregs from NOD mice.

## 3. Discussion

Previously, we generated a transgenic NOD mouse model expressing a constitutively active *Stat5b* gene in DCs (NOD.Stat5b-CA). Earlier studies have reported the generation and characterization of transgenic mice expressing constitutively active Stat5b (Stat5b-CA), but in those models, the expression was either ubiquitous or not limited to DCs [40,41,42]. In contrast, our study utilizes a tissue-specific CD11c-promoter driver to restrict Stat5b-CA expression specifically to CD11c^+^ DCs. This distinction is critical, as it enables the dissection of the cell-intrinsic effects of active Stat5b signaling in DCs, independent of its known roles in other immune cell types such as T cells, B cells, and NK cells. Our model provides a unique platform to investigate how constitutive Stat5b activation within the DC compartment influences DC differentiation, maturation, antigen presentation, and crosstalk with T cells in both homeostatic and pathological settings. Our preliminary findings indicated that expression of active STAT5b exclusively in DCs reinstated their tolerogenic properties, allowing them to re-educate Tregs and maintain long-term immune tolerance, thus protecting against T1D. Notably, we observed a significant reduction, approximately 50%, in the proportion of CD8^+^ T cells in the peripheral lymphoid organs of NOD.Stat5b-CA mice compared to NOD mice, with no change in the proportion of CD4^+^ T cells [25]. However, the precise mechanisms driving the reduction in CD8^+^ T cell frequency in NOD.Stat5b-CA mice remain unclear and require further exploration.

The present study elucidates the underlying mechanisms responsible for the observed reduction in CD8^+^ T cell frequencies in peripheral lymphoid organs of NOD.Stat5b-CA mice. Our findings suggest that this reduction is not attributable to negative selection within the thymus but rather to peripheral immune tolerance mechanisms operating within lymphoid organs. This is supported by the observed unchanged frequency of CD8^+^ T cells in the thymus of NOD.Stat5b-CA mice compared to NOD mice, while significant reductions were observed in peripheral organs. Additionally, the frequency of CD4^+^ T cells remained unaltered in both the thymus and peripheral lymphoid organs of NOD.Stat5b-CA mice, indicating the involvement of a specific regulatory mechanism affecting CD8^+^ T cells. A previous study has demonstrated that Tregs can selectively induce the apoptosis of CD8^+^ T cells but not CD4^+^ T cells in the context of certain cancers such as HNSCC [33]. Our data aligns with these findings, showing that NOD.Stat5b-CA mice had a higher frequency and total number of Tregs in the thymus and peripheral lymphoid organs than NOD mice. Additionally, these Tregs exhibited higher levels of Foxp3 expression, which is critical for their suppressive function [43,44]. The elevated expression of Foxp3 in Tregs from NOD.Stat5b-CA mice indicate an enhanced regulatory function, which could lead to more effective suppression of CD8^+^ T cell responses. This finding aligns with previous studies showing that increased Foxp3 expression correlates with stronger Treg-mediated immunosuppression [45,46].

Tregs exported from the thymus to the periphery are known to exhibit a naïve phenotype and need activation, either in vitro or in vivo, to achieve their full suppressive potential [34]. In NOD.Stat5b-CA mice, the increased expression of CD69 and OX40 and downregulation of CD62L suggests a heightened Tregs activation state. CD69, an early marker of T cell activation, has been shown to protect Tregs from immune damage and is involved in their highly suppressive function, including the induction of apoptosis in γδT cells [47]. OX40, which is critical for Tregs proliferation and survival [48], was elevated in peripheral lymphoid organs of NOD.Stat5b-CA mice, suggesting enhanced Treg stability in Stat5b-CA.DC environment. This is consistent with previous findings showing that OX40 signalling stabilizes Treg function and enhances their regulatory function [49,50]. Interestingly, the expression of CD62L, a homing receptor crucial for Treg migration, exhibited a distinct pattern between the two mouse strains. In the thymus, CD62L expression levels were comparable in both NOD and NOD.Stat5b-CA mice, suggesting that the early stages of Treg development and thymic egress are not significantly impacted by the Stat5b defect in DCs of NOD mice. In contrast, in peripheral lymphoid organs, CD62L expression was notably reduced in Tregs from NOD.Stat5b-CA mice. This differential CD62L expression aligns with findings that CD62L is essential for the homing of Tregs to peripheral lymphoid tissues, where they downregulated CD62L and exert their regulatory functions [51]. Other studies have indicated that Tregs with lower CD62L expression tend to reside more in peripheral tissues rather than circulating between lymphoid organs [52,53]. These observations are consistent with the finding of reduced CD62L expression in peripheral lymphoid organs in NOD.Stat5b-CA mice, possibly reflecting a greater retention of CD69^high^OX40^high^CD62L^low^ Tregs in peripheral tissues to exert local immune regulation. Thus, our data support the conclusion that Stat5b-CA-expressing DC promotes highly suppressor Treg, as we previously reported [25], through induction of the expression markers required for their activation, proliferation, survival, and high suppressive function.

Tregs are essential in maintaining immune tolerance and preventing autoimmune diseases. They can be categorized into central Tregs (cTregs) and effector Tregs (eTregs) based on their activation status and expression of specific cell surface markers. In mice, cTregs exhibit a CD44^low^CD62L^high^ phenotype, while eTregs are characterized by a CD44^high^CD62L^low^ phenotype [54]. eTreg cells express higher levels of CTLA-4 and ICOS compared to cTreg cells, which likely contributes to their enhanced suppressive activity [55,56]. Interestingly, T1D progression is shown to correlate with the decrease in ICOS expression by Treg infiltrated in the pancreatic islets [57]. There is increasing evidence that suggests a strong association between the occurrence of autoimmune diseases and decreased number of eTregs [58,59]. It has also been shown that tolerogenic treatment of NOD mice with B9-23 insulin peptide induces eTregs and ameliorates T1D [60]. In agreement with these observations, our data also showed that diabetes-resistant NOD.Stat5b-CA mice exhibited a lower frequency of CD44^low^CD62L^high^ cTregs and a higher frequency of CD44^high^CD62L^low^ eTregs compared to Tregs of NOD mice. Our data also highlight the important role that tolerogenic Stat5b-expressing DCs play in the reduction of cTregs and induction of eTregs subsets and protection from diabetes in NOD.Stat5b-CA mice. These findings are supported by previous studies that reported lower numbers of eTregs and higher numbers of cTregs in T1D patients, compared to control healthy groups [61]. On the contrary, another study showed that multiple sclerosis patients exhibit a reduction in cTregs and an increase in eTregs subsets as compared to healthy donors [62]. Moreover, the observed eTreg phenotypes correlate with disease activity and progression and could be a result of the resting Tregs activation in response to the autoimmune response [62]. These patterns underscore the complex roles of Treg subsets in different autoimmune diseases and the importance of their balanced regulation to restore autoimmune tolerance. However, how these heterogeneous Treg subsets are differentiated and controlled during the establishment and maintenance of immune tolerance is still unclear [60]. Okubo et al. have shown that TNFR2 agonists can trigger the conversion of resting Tregs into activated eTregs, that effectively suppress autologous cytotoxic CD8^+^ T cells [61]. Other studies suggested that eTreg generation relies more on oxidative phosphorylation relative to glycolysis [63,64]. These observations raise the need to investigate the mechanisms by which tolerogenic Stat5b-CA expressing DCs induced eTreg differentiation in the context of autoimmune tolerance which will be explored in the future in our laboratory. Thus, therapeutic strategies aimed at promoting eTreg conversion could be beneficial in managing autoimmune conditions by enhancing Treg function and stability.

Tregs suppress effector T cells through various mechanisms, including the granzyme/perforin-dependence manner [65]. The investigation of granzyme B and perforin expression in Tregs from NOD.Stat5b-CA and NOD mice align with findings on the role of these cytolytic pathways in Treg-mediated suppression [66]. Gondek et al. demonstrated that CD4^+^CD25^+^ Tregs can suppress effector T cells through a Granzyme-B-dependent but perforin-independent mechanism, indicating that granzyme B plays a crucial role in Treg-mediated suppression even in the absence of perforin [67]. On the contrary, other studies suggest that both granzyme B and perforin are involved in Treg suppressive functions. For example, Cao et al. showed that Tregs utilize granzyme B and perforin to mediate suppression in the tumor environment, where these cytolytic molecules are essential for controlling NK and CD8^+^ T cell responses [65]. In support of the dual role of these pathways in Treg suppression function, Choi et al. reported that human Tregs can use the granzyme–perforin to kill glioblastoma cells when redirected by a bispecific antibody, demonstrating the potential for granzyme B and perforin contribution to Treg-mediated cytotoxicity beyond their traditional suppressive roles [68]. Additionally, other studies also indicated that Tregs can express both granzyme B and perforin, as crucial mechanisms for their suppressive functions in autoimmune responses and cancer [32,69]. The findings in NOD.Stat5b-CA mice, showing increased frequencies and numbers of Foxp3^+^perforin^+^ and Foxp3^+^granzyme B^+^ Treg cells compared to NOD mice, corroborate these studies and suggest an enhanced cytolytic potential of Tregs in NOD.Stat5b-CA mice. This enhanced Treg cytolytic mediator expression could be crucial for the observed reduction in CD8^+^ T cell populations in NOD.Stat5b mice, further supporting our initial hypothesis.

The findings that NOD.Stat5b-CA mice exhibit a higher frequency of Foxp3^+^FASL^+^ Treg cells and elevated FAS ligand (FASL) expression on Tregs compared to NOD mice, suggesting that NOD.Stat5b-CA Tregs can also use the FAS/FASL pathway to suppress effector T cells by promoting their apoptosis. These results are supported by other studies that underscore the critical role of FASL in Treg-mediated immune regulation in various disease contexts [33,70]. In HNSCC patients, Tregs exhibit higher levels of FASL expression compared to normal controls [33]. These FASL-expressing Tregs were found capable of selectively inducing apoptosis of CD8^+^ effector T cells but not in CD4^+^ T cells, highlighting the specificity of FASL-mediated CD8^+^ T cell suppression. The involvement of the FASL pathway was confirmed by the significant reduction of CD8^+^ T cell apoptosis upon the inhibition of the FAS/FASL pathway using FASL-blocking antibody [33]. These studies suggest that FASL^+^ Tregs play a crucial role in regulating immune responses by controlling CD8^+^ T cell activity, thereby preventing excessive immune reactions that could lead to autoimmunity or tissue damage. Likewise, the critical role of FASL in Treg suppressive function was also reported in HCC patients [70]. Indeed, the authors of this study showed that Tregs expressing high levels of FASL are associated with increased apoptosis of circulating CD8^+^ T cells, suggesting that FASL^+^ Tregs contribute to the suppression of anti-tumor immunity by facilitating immune evasion and enhancing tumor growth and progression [70]. Such findings highlight the dual role of FASL^+^ Tregs in both maintaining immune tolerance and potentially aiding in tumor immune evasion. The potential therapeutic of FASL^+^ Tregs in autoimmune diseases, such as autoimmune diabetes, was highlighted by Kaminitz et al. in a study showing that the transfer of highly suppressive FASL^high^-expressing Tregs delays the onset of hyperglycemia, reduces insulitis, and protects NOD mice against T1D [71]. Consistent with the reduction of CD8^+^ T cells in the lymphoid organs of NOD.Stat5b-CA mice, Tregs from NOD.Stat5b-CA mice were found to display a significantly higher capacity to suppress the proliferation of diabetogenic 8.3-CD8^+^ T cells compared to Tregs from NOD mice. In addition, enhanced Tregs suppressive function was maintained even at a lower Treg: CD8^+^ T cell ratio, indicating a robust and superior Tregs suppressive function in NOD.Stat5b-CA. These findings are consistent with a previous report from our laboratory showing that a single injection of NOD.Stat5b-CA educated Tregs into prediabetic NOD mice reverse ongoing islet inflammation and protect NOD mice from developing diabetes [25]. Importantly, the suppressive activity of Tregs by STAT5 overexpression in DCs could enhance tumorigenicity, and therefore, beta cell antigen-specific Tregs endowed with highly suppressive function are suitable for T1D treatment. The follow-up of NOD.Stat5b-CA transgenic mice for diabetes and any sign of cancer development for more than 1 year did not show any sign of cancer. Moreover, macroscopic anatomy examination did not reveal tumor development in these mice.

A limitation of this study is the lack of direct in vivo evidence confirming the ability of Tregs to suppress the proliferation of autoreactive CD8^+^ T cells and their capacity to induce apoptosis of autoreactive diabetogenic CD8^+^ T cells in NOD.Stat5b-CA mice. Additionally, the specific cytotoxic mechanisms employed by Tregs, such as granzyme B, perforin, or the Fas/FasL pathways, remain incompletely understood. Investigating these mechanisms, potentially through approaches involving perforin inhibitors or Fas-deficient models, would provide critical insights into the molecular processes by which Tregs mediate the reduction of CD8^+^ T cells in diabetes-resistant NOD.Stat5b-CA mice.

## 4. Materials and Methods

### 4.1. Animals

NOD mice were obtained from the Jackson Laboratory (Bar Harbor, ME, USA). The NOD.Stat5b-CA transgenic mice were generated in our laboratory. Briefly, NOD.Stat5b-CA transgenic mice were generated on a NOD background at the JDRF Center of Immunological Tolerance in T1D facilities at Harvard Medical School, Boston. Briefly, the cDNA encoding wild (C57BL/6) Stat5b1*6 obtained from the vector pMX-puro-Stat5b1*6 (from Dr. Kitamura, Tokyo, Japan) was subcloned into the retroviral constructs pMYc-pIRES2-EGFP to engineer pMYc-Stat5b1*6-IRES2-EGFP. The later vector was successfully cloned into the vectors containing CD11c promoter (from Dr. K. Karjalainen, Nanyang Technological University, Singapore) to obtain the CD11c-Stat5b1*6-IRES-EGFP DNA bicistronic transgene that was used to generate NOD.Stat5b-CA mice. NOD.Stat5b-CA transgenic mice were fully protected from diabetes, whereas ≥90% of littermate control mice developed diabetes, similar to NOD mice in our colony [25]. 8.3-NOD mice, a gift from Dr. P. Santamaria at the University of Calgary (Calgary, AB, Canada), were bred in our mouse facility. All mice were maintained in a specific-pathogen-free environment with ad libitum access to food and water and under a 16 h light−8 h dark cycle at 20 ± 2 °C. For the experiments, female NOD and NOD.Stat5b-CA mice aged between 4 and 6 weeks were used. All experimental procedures adhered to the guidelines and regulations of the University of Sherbrooke’s institutional animal care committee, under protocol approval number 2022-3652.

### 4.2. Antibodies and Flow Cytometry

Mouse antibodies used in this study included anti-CD4-APC and PE (clone GK1.5; cat# 4313001, 2198944), anti-CD62L-PE-Cy7 (clone MEL-14; cat# 2548833), anti-OX40-PE (clone OX-86; cat# 12-1341-81), anti-FASL-APC (clone MFL3; cat# 2367457), anti-CD44-Alexa-eFluor 700 (clone IM7; cat# 2403352). All these antibodies were purchased from eBioscience (San Diego, CA, USA). Additional antibodies, including anti-CD8-PE (clone 53-6.7; cat# 553033), anti-CD69-Percp-Cy5.5 (clone H1.2F3; ca# 551113), and Annexin V-FITC (cat# 556419) were obtained from BD Biosciences (Mountain View, CA, USA). Single-cell suspensions for flow cytometry analysis were prepared from the thymus and peripheral lymphoid organs (spleen, mesenteric lymph nodes (MLN), and pancreatic lymph nodes (PLN) following established protocols. Specifically, tissues were harvested and mechanically dissociated using fine scissors and gentle trituration. The resulting cell suspensions were filtered through a 70 μm nylon mesh to remove debris and clumps, ensuring a single-cell preparation. For extracellular staining, cells were incubated with fluorochrome-conjugated antibodies specific to surface markers at 4 °C for 30 min in the dark. After incubation, cells were washed with phosphate-buffered saline (PBS) containing 1% bovine serum albumin (BSA) to remove unbound antibodies and reduce non-specific binding.

To evaluate the intracellular expression level of Foxp3, and production of granzyme B, and perforin, cells underwent initial staining with antibodies targeting surface markers. For granzyme B and perforin expression, cells were first incubated with brefeldin A (Cat# 00-4506-51, eBioscience, San Diego, CA, USA) and monensin (Cat# 420701, Bilegend, San Diego, CA, USA) for 4 h. Thereafter, cells were washed with PBS, fixed, and permeabilized with Foxp3 fixation/permeabilization staining buffer (Cat# 00-5523-00, eBioscience, San Diego, CA, USA) for 45 min. Subsequently, intracellular staining was performed using a monoclonal antibody specific to Foxp3-FITC (clone FJK-16s; cat# 2007700) anti-Perforin-APC (clone eBOMAK-D; cat# 2384190), and anti-Granzyme B-PE-Cy7 (clone NGZB; cat# 2514099) at 4 °C for 45 min. All these antibodies were purchased from eBioscience (San Diego, CA, USA). The cells were then washed with PBS containing 1% BSA. Finally, samples were resuspended in PBS for analysis on a flow cytometer. Cell acquisition was performed on a CytoFLEX instrument (Beckman Coulter, Brea, CA, USA), and data were analyzed using FlowJo 10.2 software (Tree Star Inc., Ashland, OR, USA).

### 4.3. Memory and Effector Treg Analysis

Single-cell suspensions from the spleen were prepared as described above. Briefly, 5 × 10^5^ cells per well were resuspended in RPMI 1640 (Life Technologies, Carlsbad, CA, USA) supplemented with 10% FBS (Gibco, Waltham, MA, USA), 2 mM L-glutamine, 0.1% penicillin/gentamicin, and 50 mM β-Mercaptoethanol. Cells were then either left unstimulated or stimulated with plate-bound anti-CD3 mAbs (5 μg/mL) and anti-CD28 (2 μg/mL), together with IL-2 (20 U/mL) and incubated for 24 h at 37 °C, 5% CO_2_ incubator. Cells were harvested, washed with PBS, and effector and memory Tregs were analyzed by staining cells with mAbs against CD4, CD25, CD62L, and CD44 and combined with intracellular staining with anti-Foxp3 mAbs. After staining, cells were washed and acquired using a Cytoflex instrument (Beckman Coulter, Brea, CA, USA), and analyzed with FlowJo software (Tree Star, Ashland, OR, USA).

### 4.4. Treg Purification and In Vitro Suppression Assay

CD4^+^CD25^+^ T cells (Tregs) were purified from the spleens of NOD and NOD.Stat5b-CA mice using a Treg isolation kit (Miltenyi Biotec, San Diego, CA, USA), following the manufacturer’s protocol and previously published methods [25]. The purified cells were checked for Treg cell purity, which was ˃92%.

CD8^+^ T cells were isolated from the spleens of 8.3-NOD mice, a model that expresses a highly diabetogenic T-cell receptor (TCR) specific for an islet antigen, facilitating studies on autoimmune diabetes [72]. The isolated CD8^+^ T cells were then labeled with 5 μM CFSE [(CellTrace™ Carboxyfluorescein diacetate, succinimidyl ester (CFSE) Cell Proliferation Kit; Molecular probes by Life Technologies, Carlsbad, CA, USA)], a fluorescent cell-staining dye used to monitor cell division by flow cytometry, as described previously [73]. In brief, CD8^+^ T cells were labeled with CFSE and placed in a dark environment at 37 °C for 10 min. To terminate the labeling process, an equal volume of T-cell medium supplemented with 20% heat-inactivated fetal bovine serum (FBS) (PAA, Cölbe, Germany) was added, and the cells were incubated for an additional 20 min. The cells were then washed four times with a T-cell medium to remove any excess dye.

To evaluate the suppressive function of Tregs, purified Tregs (2 × 10^4^ cells/well) from NOD and NOD.Stat5b-CA mice were co-cultured with anti-CD3 (mAb) (5 μg/mL) and anti-CD28 mAb (2 μg/mL)-stimulated CFSE-labeled 8.3-CD8^+^ T cells at different ratios (1:1, 1:2, and 1:4) in a 96-well culture plate. CD8^+^ T cells cultured alone without Tregs were used as a control. After a 3-day incubation period at 37 °C, cells were harvested and stained with an anti-CD8-APC antibody to identify CD8^+^ T cells, and their proliferation was then assessed by measuring the dilution of CFSE fluorescence using a Cytoflex instrument (Beckman Coulter, Brea, CA, USA) and analyzed by FlowJo 10 (Tree Star, Ashland, OR, USA) software.

### 4.5. Apoptosis Assay

Purified Tregs from NOD and NOD.Stat5b-CA mice were co-cultured with 8.3-CD8^+^ T cells at different ratios as described in the suppression assay method. After 24 h, the cells were harvested and stained with anti-CD8-APC antibody, FITC-labeled Annexin V (BD Biosciences), and propidium iodide (PI) (BD Biosciences). The cells were then incubated for 30 min at room temperature in the dark and analyzed using a Cytoflex instrument (Beckman Coulter, Brea, CA, USA). FACS data were processed with FlowJo software (Tree Star, Ashland, OR, USA).

### 4.6. Statistical Analysis

Data were analyzed using the GraphPad Prism 10.0 software (GraphPad Software Inc., La Jolla, CA, USA) and presented as the mean ± SEM. Student’s *t*-test or one-way ANOVA followed by Tukey’s post hoc test were used to calculate *p* values. *p* ˂ 0.05 was determined to be significant. All experiments were repeated three to five times.

## 5. Conclusions

Our study demonstrates that the expression of active Stat5b in DCs of NOD mice reinstates their ability to generate highly suppressive Tregs and also results in a marked reduction in the proportion of effector CD8^+^ T cells in peripheral lymphoid organs, without affecting CD4^+^ T cell development. This reduction in CD8^+^ T cells is attributed to enhanced Treg-mediated suppression in the periphery, rather than thymic selection. Moreover, our study also shows increased effector CD44^high^CD62L^low^ Tregs population, as well as elevated granzyme B, perforin, and FAS ligand expression in Tregs, underlying the multifaceted mechanisms by which highly suppressive Tregs mediate immune suppression in diabetes-resistant NOD.Stat5b-CA mice. These findings could be exploited for a better design of Treg-based therapy in T1D and other autoimmune diseases.

## Data Availability

The datasets generated during and/or analyzed during the current study are available from the corresponding author upon reasonable request.

## Supplementary Materials

The following supporting information can be downloaded at: https://www.mdpi.com/article/10.3390/ijms27020794/s1.
