# Supplementary material for: Constitutively Active Stat5b Expression in Dendritic Cells Enhances Treg-Mediated Elimination of Autoreactive CD8+ T Cells in Autoimmune Diabetes
Source: Int J Mol Sci. 2026 Jan 13;27(2):794. doi: 10.3390/ijms27020794 (PMC12841221; doi:10.3390/ijms27020794)
Supplement: Supplementary file 1 [file ijms-27-00794-s001.zip › ijms-3558079-supplementary.pdf]

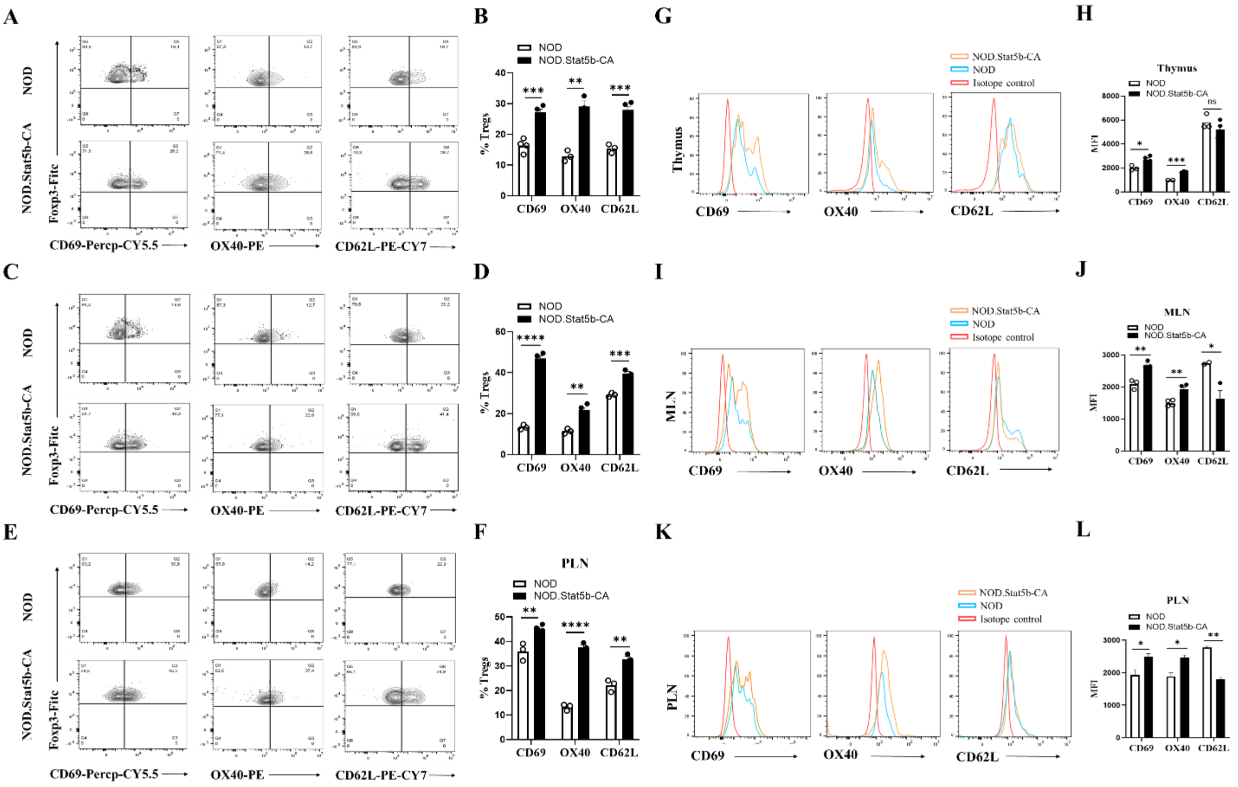

Supplementary Figure S1. Tregs of NOD.Stat5b-CA mice exhibit an activated phenotype. (**A**–**F**) Dot plots and bar graphs showing Foxp3$^+$CD69$^+$, Foxp3$^+$OX40$^+$, and Foxp3$^+$CD62L$^+$ cell frequencies among the CD4$^+$Foxp3$^+$ T cell population in the thymus, MLN, and PLN of NOD and NOD.Stat5b-CA mice. (**G**–**L**) Representative histograms (left side) and quantification of Mean Fluorescence Intensity (MFI) values (right side) showing CD69, OX40, and CD62L expression levels in CD4$^+$Foxp3$^+$ Tregs in NOD and NOD.Stat5b-CA mice. Error bars indicate the mean ± SEM of three-four independent experiments (n = 4). *p*-values were calculated using the student's *t*-test. * $p < 0.05$; ** $p < 0.01$; *** $p < 0.001$; **** $p < 0.0001$.
